# The role of bone morphology of the greater tuberosity and lateral acromion on subacromial space during scaption: a three-dimensional dynamic simulation analysis

**DOI:** 10.1186/s12891-023-06957-y

**Published:** 2023-11-15

**Authors:** Rodrigo Brandariz, Caecilia Charbonnier, Alejandro Culebras Almeida, Alexandre Lädermann, Gregory Cunningham

**Affiliations:** 1 Shoulder and Elbow Center La Colline, Geneva, Switzerland; 2 Medical Research Department, Artanim Foundation, Meyrin, Switzerland; 3 Faculty of Medicine, University of Geneva, Geneva, Switzerland; 4 Department of Orthopaedics and Traumatology, Réseau Hospitalier Neuchâtelois, Neuchâtel, Switzerland; 5 Division of Orthopaedics and Trauma Surgery, La Tour Hospital, Meyrin, Switzerland; 6 Division of Orthopaedics and Trauma Surgery, Department of Surgery, Geneva University Hospitals, Geneva, Switzerland

**Keywords:** Greater tuberosity angle, Critical shoulder angle, Subacromial space narrowing, Shoulder scaption, Dynamic CT scan, 3D motion capture

## Abstract

**Background:**

The bone morphology of the greater tuberosity and lateral acromion plays a central role in subacromial impingement syndrome. The critical shoulder angle (CSA) and greater tuberosity angle (GTA) are two-dimensional measurement parameters that have been validated to evaluate it radiologically. These markers are, however, static and don’t consider the dynamic effect of glenohumeral motion.

**Objectives:**

This study aimed to better understand the biomechanics in subacromial impingement with a dynamic simulation based on a validated 3D biomechanical model coupling joint kinematics and 3D reconstructed computed tomography.

**Study design & methods:**

Sixty-one patients were included in this study: a case group of 44 patients with degenerative rotator cuff tears involving only the supraspinatus, and a control group of 17 without a rotator cuff tear. Patients with previous surgeries, traumatic cuff tears, and cuff tear arthropathy were excluded. CSA, GTA, and impingement-free range of motion (IF-ROM) of the glenohumeral joint in scaption were calculated. Correlation tests were used to determine the relationship between ROM and CSA, GTA, and combined CSA and GTA values.

**Results:**

CSA and GTA were significantly higher in the rotator cuff tear group (p = 0.001 and < 0.001), while IF-ROM was significantly higher in the control group (p = 0.001). There was no overall correlation between CSA and GTA (R = 0.02, p = 0.8). Individual correlation between both angles with IF-ROM was negatively weak for CSA (R = -0.4, p < 0.001) and negatively moderate for GTA and IF-ROM (R = -0.5, p < 0.001). However, combining both angles resulted in a negatively high correlation with IF-ROM (R = -0.7, p < 0.001).

**Conclusion:**

Subacromial space narrowing during scaption is highly correlated to the cumulative values of GTA and CSA. These findings suggest that the combined bony morphology of the lateral acromion and greater tuberosity plays an important role in subacromial impingement.

**Level of evidence:**

III

## Background

Subacromial pain syndrome represents the main shoulder complaint and the reason for consultation and sick leave [1]. It involves a spectrum of pathologies, including rotator cuff tendinosis, calcific tendinitis, subacromial bursitis and partial or full-thickness rotator cuff tears (RCT) [31]. Concerning this last entity, two main theories have been postulated that attempt to explain the physiopathogenesis of these lesions.

On the one hand, some authors describe the intrinsic theory based on the idea that an intrinsic (internal) process causes the tear, such as degeneration of the rotator cuff tendon, rather than an external compression. It suggests that the tear is caused by repetitive microtrauma and a gradual tendon breakdown due to ageing and wear and tear rather than an acute injury or trauma [22, 34]. Many genetic, metabolic, and behavioral factors are thought to contribute to intrinsic tendon tearing [21]. On the other hand, the extrinsic theory suggests that the rotator cuff is subject to external stresses, such as traction and tendon compression in the subacromial space (impingement). The term subacromial impingement was thought to result from an accumulation of bursal fluid in the shoulder joint. It was not until the 1970s that Charles Neer developed the concept of rotator cuff impingement, suggesting that the rotator cuff and long head of the biceps tendon were being impinged between the humerus and the acromion [25]. Since then, advances in medical imaging technology have allowed further investigation of shoulder impingement and a better understanding of the anatomy of the shoulder joint and the role of the rotator cuff and biceps tendon in shoulder impingement [16, 25]. RCT could be due to direct contact of the tendon against the bone or indirectly due to a change in muscle force vectors induced by lateral acromion morphology [13].

**Fig. 1:**
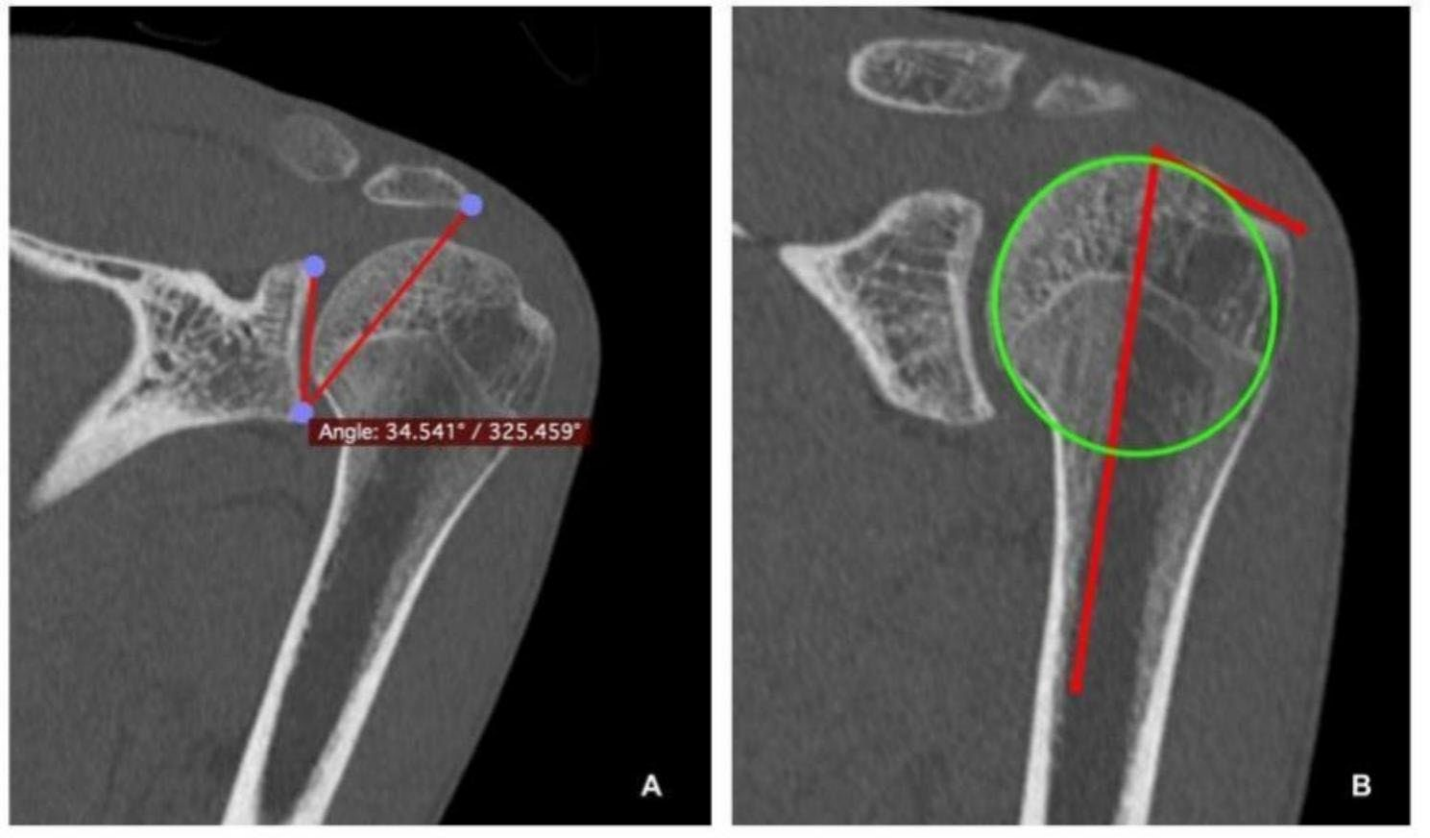
3D corrected CT-scan reconstruction of a left shoulder showing the assessment of CSA and GTA. (**A**) CSA is based on the angle between the glenoid plane and most lateral border of the acromion. (**B**) GTA consists of the angle between a parallel line to the diaphyseal axis that passes through the humeral head center of rotation and the most superolateral edge of the greater tuberosity.

Nevertheless, to reduce subacromial space, it should be narrowed with, at least, two structures, meaning that the greater tuberosity may also play an important role in the development of impingement. Consequently, not only the acromion but also the greater tuberosity could be associated with RCT [19, 26, 27]. Under this perspective, recent studies have proposed and validated the critical shoulder angle [24] (CSA) and greater tuberosity angle [9] (GTA) as radiographic markers associated with RCT. CSA takes into account the lateral extent of the acromion according to the glenoid plane, whereas GTA takes into consideration the superolateral extent of the greater tuberosity according to the humeral head center of rotation (Fig. 1). However, both parameters are static, and may not represent the dynamic process of shoulder impingement during shoulder motion [9, 24].

For these reasons, this study aimed to propose a better understanding of the biomechanics of subacromial impingement with a dynamic simulation based on a validated three-dimensional (3D) biomechanical model coupling joint kinematics and 3D reconstructed computed tomography (CT).

## Materials and methods

### Patients recruitment and groups

We prospectively recruited all patients with at least 3-tesla magnetic resonance imaging (MRI) presenting to a shoulder-specialized institute between 2018 and 2022. Patients who presented a degenerative rotator cuff tear involving only the supraspinatus were included in the case group (RctG), whereas those without a supraspinatus tear were included in the control group (CoG). Patients with previous history of shoulder surgery or any grade of arthropathy were excluded. The study protocol was approved by the local ethics committee (AMG-12.18), and all patients gave informed written consent.

### 3D reconstruction and CSA/GTA measurements

All patients underwent a CT of the shoulder. The CT examinations were conducted with a LightSpeed (LS) VCT 64 rows (General Electric Healthcare, Milwaukee WI, USA). Images were acquired at 0.63 mm slice resolution. Based on the CT images, patient-specific 3D models of the shoulder bones (scapula and humerus) were reconstructed for each patient using Mimics software (Materialize NV, Leuven, Belgium).

Morphological measurements were performed to analyze individual shoulder anatomy. Both angles (CSA and GTA) were measured on 3D corrected coronal CT slicing as validated by Bouaicha et al., [2] by two independent observers (GC, LACA), using Osirix (Pixmeo, Bernex, Switzerland) (Fig. 1). All measures were repeated twice by each of the observers, with a time interval of two weeks in between, and the mean values were taken for the statistical analysis.

### Kinematics analysis and impingement detection

Using the reconstructed patient-specific 3D models of the shoulder bones (scapula and humerus), biomechanical parameters were first computed to allow motion description of the glenohumeral joint. The glenohumeral joint center was calculated by a sphere fitting technique [29] that fits a sphere to the humeral head model. Bone coordinate systems were established for the scapula and humerus based on the definitions suggested by the International Society of Biomechanics [37] using anatomical landmarks defined on the bone models.

**Fig. 2:**
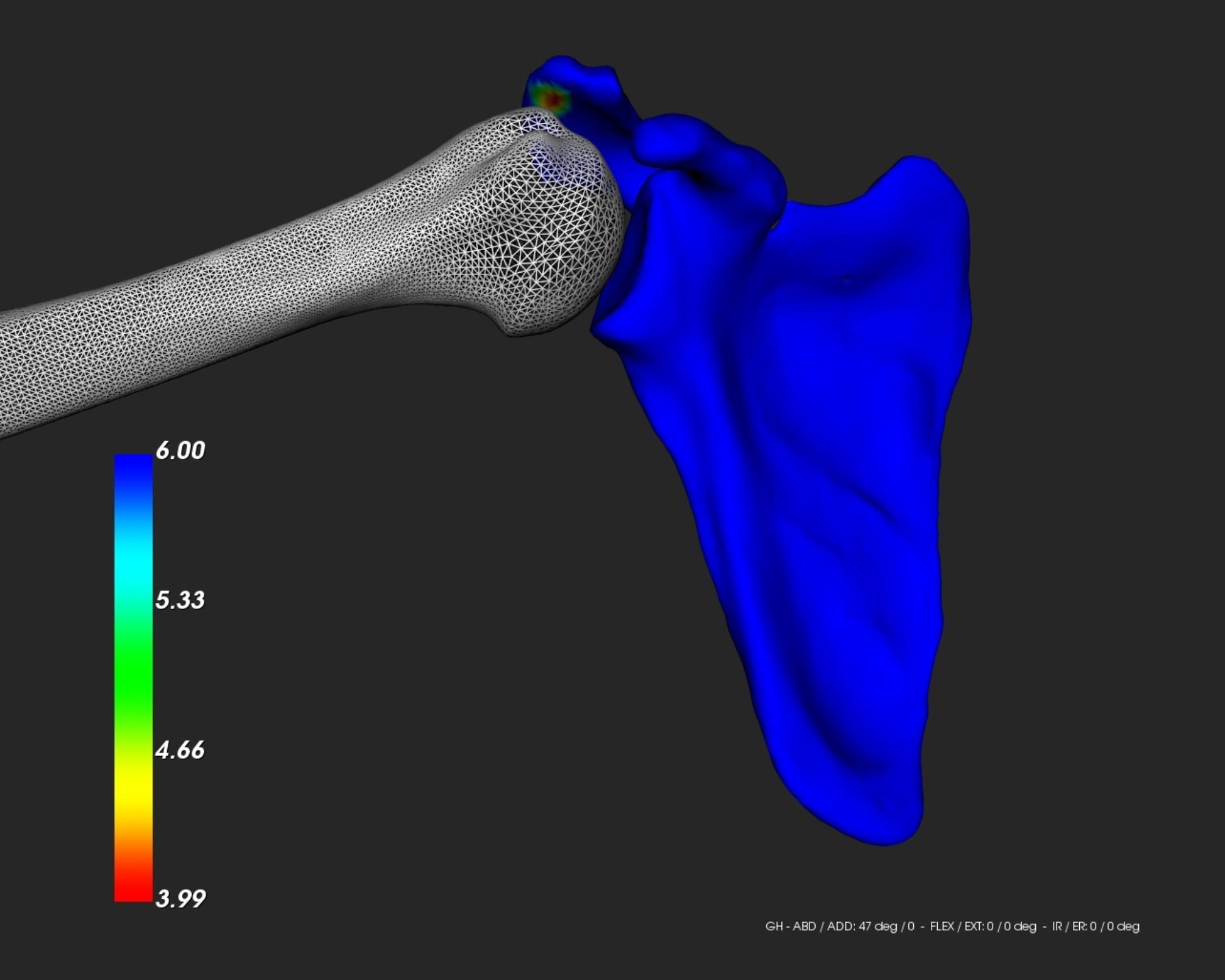
Visualization of the acromio-humeral distance during scaption

Second, the shoulder ROM was applied at each time step to the humerus model in its anatomical frame with real-time evaluation of impingement, using a dedicated simulation software [4] that integrates a dynamic module allowing shoulder joint kinematics computation from standard kinematic sequences (e.g., elevation, scaption, internal/external rotation). This is achieved as follows: the humerus model is simulated by increasing the relevant rotational angle of 1° at each time step taking into account the patient-specific biomechanical parameters of the joint (glenohumeral joint center and anatomical planes, i.e., anatomical frame), whereas the scapula model remains fixed at all time. In the present study, we were interested in simulating a pure scaption motion only, ranging from 0° to 120°. During motion simulation, the minimum acromio-humeral distance (AHD) that is typically used for the evaluation of subacromial impingement was measured at each time step [7, 15, 33]. This distance was calculated in millimeters based on the simulated bone models’ positions [5]. A color scale was also used to map the variations of distance on the scapula surface (red color = minimum distance, other colors = areas of increased distance), as shown in Fig. 2. Given the thickness of the potential impinged tissues, subacromial impingement was considered when the computed AHD was < 6 mm, as suggested in the literature [5, 7, 10]. At the end of each simulation, the IF-ROM during the scaption was thus recorded.

*Visualization of the acromio-humeral distance during scaption. The colors represent the variations of the distance between the acromion and humeral head with the red color denoting the zone of minimum distance.*

### Statistical analysis

Statistical analyses were performed using Stata 16 software (StataCorp, College Station, TX, USA). Baseline patient characteristics were compared using χ2 tests of proportions (ratios) and Student t tests according to the characteristic of each variable. The Shapiro-Wilk test was used to evaluate the distribution of the sample. Inter- and intraobserver reproducibility were analyzed for the measurements of the CSA and GTA values. Finally, Pearson (r) was used to determine a correlation between CSA and GTA, whereas Spearman (R) was used to determine the relationship between ROM and CSA, GTA, and combined CSA and GTA values. Statistical significance was set at P < 0.05.

## Results

Sixty-one patients were included in this study: 44 had degenerative supraspinatus tear (RctG) and 17 had no signs of cuff pathology (CoG). The affected side and gender were similar between the two groups. CSA and GTA were significantly higher in the RctG (41.3° ± 5.4 and 73.9° ± 4.4, respectively) than in the CoG (CSA 36.8 ± 4.1 and GTA 68.3 ± 3.5, p < 0.001). Moreover, the sum of these two variables was also statistically significantly higher in the first group (115.1 ± 6.2 vs. 105.1 ± 4.9, p < 0.001). The analyses of intra- and interobserver reproducibility showed almost perfect agreement for CSA and GTA (ICC being between 0.81 and 0.93 respectively). In addition, IF-ROM was significantly higher in the CoG than RctG (51.0 ± 32.2 vs. 24.3 ± 28.4, p = 0.001) (Table 1).

**Table 1:** Patient parameters and greater tuberosity angle values are compared in both groups

Variable	Patient Group (CaG) (n = 44)	Control Group (CoG) (n = 17)	P value*
Gender (n, %)			0.91
Male	26 (59)	11 (64)	
Female	18 (41)	6 (36)	
Affected side (N)			
Right	25	10	0.88
Left	19	7	
CSA (°)	41.3 ± 5.4 (29.8–52.5)	36.8 ± 4.1 (29.2–44.8)	0.001
GTA (°)	79.3 ± 4.4 (61.2–84.9)	68.3 ± 3.5 (59.0–74.3)	< 0.001
CSA + GTA (°)	115.1 ± 6.2 (102.5–130.6)	105.1 ± 4.9 (95.8–111.5)	< 0.001
IF-ROM (°)	24.3 ± 28.4 (1.0–120.0)	51.0 ± 32.2 (3.0–120.0)	0.001

CSA, Critical Shoulder Angle; GTA, Greater Tuberosity Angle; IF-ROM, Impingement free range of motion. Continuous data are shown as mean ± standard deviation (range). *Paired T-Test analysis and chi2 according to the sample

**Fig. 3:**
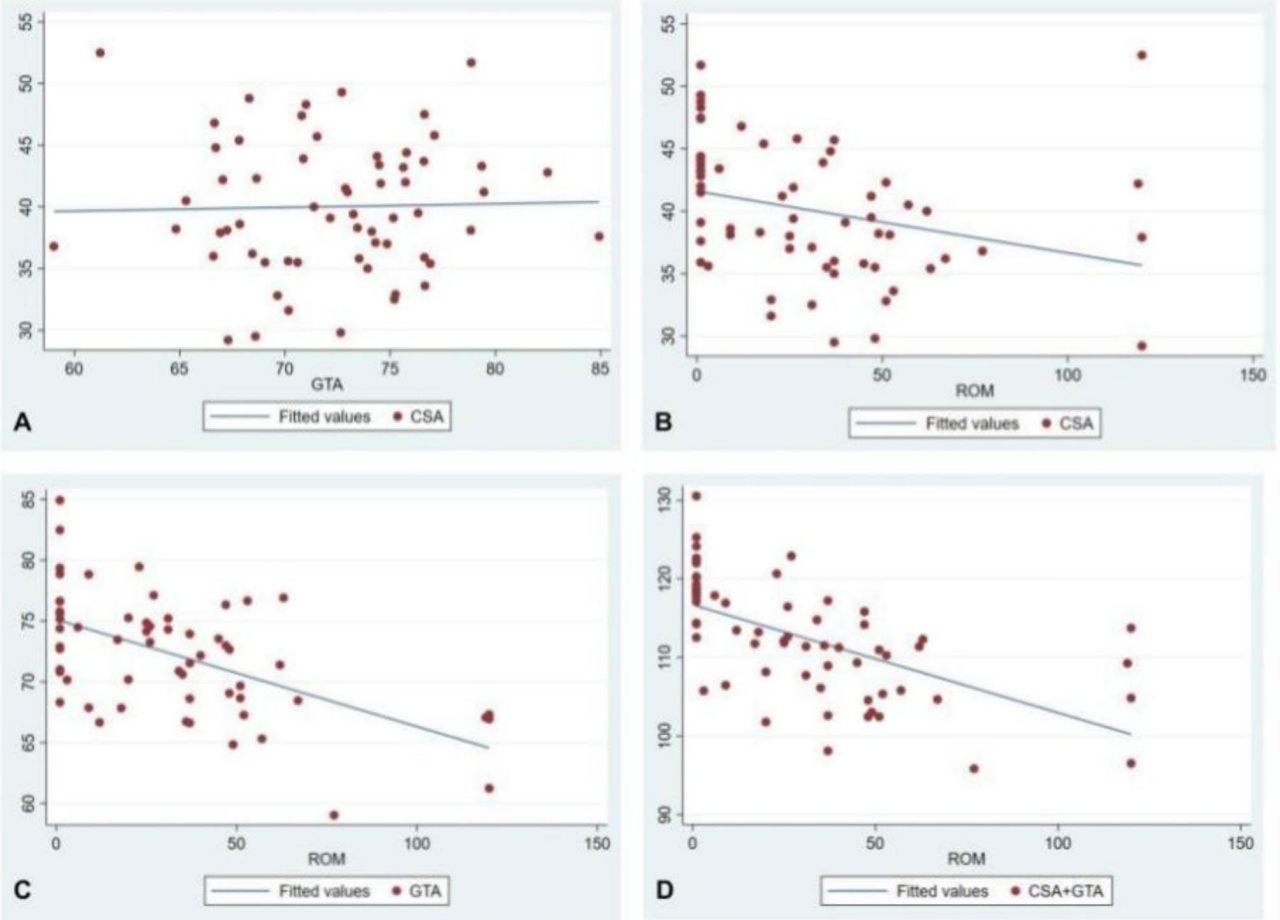
Scatter diagrams of correlation among the outcome variables. Scatter diagrams showing: (**A**) no correlation between the critical shoulder angle (CSA) and greater tuberosity angle (GTA) (R = 0.02, p = 0.8). (**B**) weak negative correlation between CSA and IF-ROM (R = -0.4, p < 0.001). (**C**) moderate negative correlation between GTA and IF-ROM (R = -0.5, P < 0.001). (**D**) high correlation between combined values of CSA and GTA with IF-ROM (R = -0.7, p < 0.001)

Overall, there was no correlation between CSA and GTA (R = 0.02, p = 0.8). The individual correlation between both angles and the IF-ROM was negatively weak to moderate (R = -0.4, p < 0.001 for CSA, and R = -0.5, p < 0.001 for GTA). However, the sum of both angles was highly negatively correlated with the IF-ROM (R = -0.7, p < 0.001) (Table 2; Fig. 3).

**Table 2:** Correlation test among the outcome variables

Variables	Correlation	P value
CSA vs. GTA	0.02	0.83*
CSA vs. ROM	-0.44	0.0004**
GTA vs. ROM	-0.52	< 0.0001**
CSA + GTA vs. IF-ROM	-0.7	< 0.0001**

CSA: Critical Shoulder Angle; GTA: Greater Tuberosity Angle; IF-ROM: Impingement-free range of motion. * Pearson correlation test. ** Spearman correlation test

## Discussion

The main findings of our study were that the combination of the CSA and GTA of the shoulder was strongly correlated with IF-ROM. However, the isolated correlation between CSA and GTA with IF-ROM was weak to moderate, respectively. This suggests that dynamic subacromial space narrowing during scaption is dependent on both morphologies of the greater tuberosity and the lateral acromion. CSA and GTA were significantly higher in the RctG group, and there was no direct correlation between both markers, corroborating the findings of previous studies [8, 9]. Cunningham et al. found that a cumulative value of GTA and CSA over 103° was associated with an over 97-fold risk of association to degenerative RCT [8, 9]. These findings point out that, apart from the static implication of both angles in the physiopathogenesis of subacromial impingement and rotator cuff tendinopathy, there is a dynamic abutment that generates more narrowing during scaption.

The implication of bone morphology and particularly CSA and GTA is not fully understood. An alteration of the subacromial space may result in direct narrowing, leading to impingement and rotator cuff tear, or it could result in a change in the force vectors of the rotator cuff muscles. Gerber et al. described in their biomechanical study that larger CSA increases the ratio of joint shear to joint compression forces, requiring substantially increased compensatory supraspinatus loads [13]. This could also be the case for GTA. Indeed, even though GTA has not been the subject of a biomechanical study such as the CSA, values ​​greater than 70° and 35° respectively, have been shown to have a positive predictive value for the presence of RCT [9, 24, 32, 38]. Further biomechanical studies such as Gerber’s should thus be carried out to investigate the relationship between the GTA and the supraspinatus vector force changes.

Ultimately, CSA has been objecting to debate related to the inaccuracies in the radiographic measurements. Chalmers et al., [3] for instance, obtained only a small difference in CSA values ​​in patients with RCT compared to their control group (34° ± 4° versus 32° ± 4°; mean difference, 2.0°; 95% CI, 0.7°-3.2°; p = 0.003). They suggested that differences as small as 2° may be easily subject to measurement mistakes. In contrast, several studies have shown strong intra- and inter-observer reproducibility for both variables (CSA and GTA) [6, 9, 30, 32].

Another debate on measurements is about the AHD. A systematic review of the literature by Mc. Creesh et al. [23] showed strong reliability for an ultrasound to measure AHD but moderate for MRI and CT scans. However, these studies only performed monoplane measurements of the subacromial space. As a consequence, Kocadal et al. analyzed the subacromial volume by measuring the AHD of the entire extension of the inferior acromion in the coronal plane of MRI, and not sagittal as previous studies have done. Thus, they showed how the volume of the subacromial space was significantly lower in 26 patients with impingement syndrome compared to the control group of 24 asymptomatic patients (3.18 ± 0.99 cm3 vs. 3.93 ± 1.06 cm3, p < 0.01) [18].

In addition, the dynamic implication of shoulder position could also lead to several biases. In 2008, Fehringer et al. warned that AHD measurement was highly variable according to the position of the shoulder at the moment of the radiography [11]. Finally, as described by Sanguanjit et al., the effect of gravity on the shoulder with decubitus changes should not be ruled out. In their study, they showed a significant decrease in AHD in supine patients compared to standing patients [28]. To reduce all possible confounders, our simulation established standardized and reproducible bone coordinate systems for the scapula and humerus based on the ISB definitions to input shoulder ROM [37], and computed the AHD in 3D at each time step over the entire scapula surface [5].

Considering the findings, the use of tuberoplasty and acromioplasty to reduce impingement could be argued to avoid recurrences. In a prospective randomized study, Lädermann et al. showed that acromioplasty without tuberoplasty removed 50% of the estimated volume of impinging acromial bone [20]. However, the role of acromioplasty in preventing tear recurrence remains open to debate. A randomized clinical trial by WaTerman et al. [35] recently showed the same long-term (mean 7.5 years) clinical outcomes and recurrence rates among patients who underwent a RCT repair with or without acromioplasty. On the contrary, Woodmass et al. reported after an average of 11 years no differences in patient-reported outcomes but a significantly higher reoperation rate in patients who had rotator cuff repair without acromioplasty [36]. Nevertheless, neither CSA nor GTA was measured in these two studies, nor was the amount of bone removed by the acromioplasty. Indeed, recent studies demonstrated that acromioplasty does not normalize CSA in more than 35% of the cases [14, 17]. However, none of these studies have looked at the effect of GTA and retear. Therefore, the clinical implication of an increase in GTA after a RCT repair remains under scrutiny. In effect, a study showed no significant differences in the results of the Constant score and Oxford shoulder score between two groups of patients with GTA above and below 70°. However, this study presents several biases due to its retrospective nature (it starts from different basal functional scores between groups, there is no imaging follow-up of tendon integrity, and they present a short follow-up for this pathology) [12]. Since both acromion and greater tuberosity morphology are implicated in shoulder impingement syndrome and RCT, further research is needed to evaluate the combined impact of acromioplasty and tuberoplasty on CSA and GTA and correlate it with long-term clinical outcomes and retear rates.

This article is not exempt from limitations. Firstly, since we considered it unethical to perform an unnecessary study on a healthy population, we enrolled patients with, for example, shoulder instability or SLAP lesions as a control group; thus, the age could vary between the group yet the prevalence of each pathology is different. However, a previous study found no correlation between age and CSA or GTA, suggesting that both parameters are constitutionally acquired, therefore, due to the fact that our study only aimed to analyze bone morphology in a simulation test we did not consider the age as an exposition variable [8]. Although this was a 3D-model analysis, GTA and CSA remain 2D markers. They are projections of the most prominent extent of the bone in the coronal plane but do not indicate precisely where the impingement takes part. This may explain why Hardy et al. found that anterior acromioplasty could affect CSA values [17]. Furthermore, other dynamic factors such as scapula dyskinesis may predispose to subacromial impingement. In addition, we used AHD to define subacromial impingement, and, as discussed previously, it may not be accurate enough to precisely determine the thickness of the impinging soft tissues because of the number of biases in the measurements according to the position of the arm, gravity force and range of movement. However, thanks to this validated biomechanical model and software, we tended to minimize them. Future studies should also envision to simulate the joint based on motion capture data instead of simplified motion such as the pure scaption used in this study to comprehend the subtle motion of the joint in the three anatomical planes and the interplay between the orientation of the humerus with respect to the scapula. Finally, since this is the first study that analyzed the dynamic behavior of the CSA and GTA during scaption, we could not assess a power analysis due to the lack of previous data in this field. Therefore, we encourage readers to roll out future studies according to our findings.

## Conclusion

Subacromial space narrowing during scaption is highly correlated to the cumulative value of GTA and CSA. These findings suggest that the combined bony morphology of the lateral acromion and greater tuberosity plays an important role in subacromial impingement.

## Data Availability

All data generated or analyzed during this study are included in this published article.

## References

[1] Baumann A, Morgan B. Subacromial Impingement Syndrome [Internet]. Diagnostic Clusters in Shoulder Conditions. 2017;57–63. 10.1007/978-3-319-57334-2_5

[2] Bouaicha S, Ehrmann C, Slankamenac K, Regan WD, Moor BK. Comparison of the critical shoulder angle in radiographs and computed tomography. Skeletal Radiol. [Internet]. 2014;43(8):1053–1056. 10.1007/s00256-014-1888-4 10.1007/s00256-014-1888-4 24744014

[3] Chalmers PN, Salazar D, Steger-May K, Chamberlain AM, Yamaguchi K, Keener JD. Does the Critical Shoulder Angle Correlate With Rotator Cuff Tear Progression? Clin. Orthop. Relat. Res. [Internet]. 2017;475(6):1608–1617. 10.1007/s11999-017-5249-1 10.1007/s11999-017-5249-1 PMC5406338 28120293

[4] Charbonnier C, Chagué S, Kevelham B, Preissmann D, Kolo FC, Rime O et al. ArthroPlanner: a surgical planning solution for acromioplasty. Int. J. Comput. Assist. Radiol. Surg. [Internet]. 2018;13(12):2009–2019. 10.1007/s11548-018-1707-9 10.1007/s11548-018-1707-9 29427059

[5] Charbonnier C, Chagué S, Kolo FC, Lädermann A. Shoulder motion during tennis serve: dynamic and radiological evaluation based on motion capture and magnetic resonance imaging. Int. J. Comput. Assist. Radiol. Surg. [Internet]. 2015;10(8):1289–1297. 10.1007/s11548-014-1135-4 10.1007/s11548-014-1135-4 25503926

[6] Cherchi L, Ciornohac JF, Godet J, Clavert P, Kempf J-F. Critical shoulder angle: Measurement reproducibility and correlation with rotator cuff tendon tears. Orthop. Traumatol. Surg. Res. [Internet]. 2016;102(5):559–562. 10.1016/j.otsr.2016.03.017 10.1016/j.otsr.2016.03.017 27238292

[7] Chopp JN, Dickerson CR. Resolving the contributions of fatigue-induced migration and scapular reorientation on the subacromial space: an orthopaedic geometric simulation analysis. Hum. Mov. Sci. [Internet]. 2012;31(2):448–460. 10.1016/j.humov.2011.09.005 10.1016/j.humov.2011.09.005 22230714

[8] Cunningham G, Cocor C, Smith MM, Young AA, Cass B, Moor BK. Implication of bone morphology in degenerative rotator cuff lesions: A prospective comparative study between greater tuberosity angle and critical shoulder angle. Orthop. Traumatol. Surg. Res. [Internet]. 2022;108(2):103046. 10.1016/j.otsr.2021.103046 10.1016/j.otsr.2021.103046 34487909

[9] Cunningham G, Nicodème-Paulin E, Smith MM, Holzer N, Cass B, Young AA. The greater tuberosity angle: a new predictor for rotator cuff tear. J. Shoulder Elbow Surg. [Internet]. 2018;27(8):1415–1421. Available from: https://linkinghub.elsevier.com/retrieve/pii/S1058274618301575doi:10.1016/j.jse.2018.02.051 10.1016/j.jse.2018.02.051 29703680

[10] De Maeseneer M, Van Roy P, Shahabpour M. Normal MR imaging anatomy of the rotator cuff tendons, glenoid fossa, labrum, and ligaments of the shoulder. Radiol. Clin. North Am. [Internet]. 2006;44(4):479–87, vii. 10.1016/j.rcl.2006.04.002 10.1016/j.rcl.2006.04.002 16829244

[11] Fehringer EV, Rosipal CE, Rhodes DA, Lauder AJ, Puumala SE, Feschuk CA et al. The radiographic acromiohumeral interval is affected by arm and radiographic beam position. Skeletal Radiol. [Internet]. 2008;37(6):535–539. 10.1007/s00256-008-0467-y 10.1007/s00256-008-0467-y 18343920

[12] Gatot C, Lee M, Chen JY, Fu Hong BA, Tijauw Tjoen DL. Increased preoperative greater tuberosity angle does not affect patient-reported outcomes postarthroscopic rotator cuff repair. JSES Int [Internet]. 2021;5(1):72–76. 10.1016/j.jseint.2020.10.008 10.1016/j.jseint.2020.10.008 PMC7846682 33554168

[13] Gerber C, Snedeker JG, Baumgartner D, Viehöfer AF. Supraspinatus tendon load during abduction is dependent on the size of the critical shoulder angle: A biomechanical analysis [Internet]. Journal of Orthopaedic Research. 2014;32(7):952–957. 10.1002/jor.22621 10.1002/jor.22621 24700399

[14] Girard M, Colombi R, Azoulay V, Laumonerie P, Martel M, Mansat P, Does anterior acromioplasty reduce critical shoulder angle? [Internet]., Orthopaedics et al. & Traumatology: Surgery & Research. 2020;106(6):1101–1106. 10.1016/j.otsr.2020.04.013 10.1016/j.otsr.2020.04.013 32703718

[15] Graichen H, Hinterwimmer S, von Eisenhart-Rothe R, Vogl T, Englmeier K-H, Eckstein F. Effect of abducting and adducting muscle activity on glenohumeral translation, scapular kinematics and subacromial space width in vivo. J. Biomech. [Internet]. 2005;38(4):755–760. 10.1016/j.jbiomech.2004.05.020 10.1016/j.jbiomech.2004.05.020 15713296

[16] Gumina S. Rotator Cuff Tear: Pathogenesis, Evaluation and Treatment [Internet]. Springer; 2016. Available from: https://play.google.com/store/books/details?id=NBZ_DQAAQBAJ

[17] Hardy V, Rony L, Bächler J, Favard L, Hubert L. Does isolated arthroscopic anterior acromioplasty modify critical shoulder angle? Orthop. Traumatol. Surg. Res. [Internet]. 2022;108(2):103040. 10.1016/j.otsr.2021.103040 10.1016/j.otsr.2021.103040 34389495

[18] Kocadal O, Tasdelen N, Yuksel K, Ozler T. Volumetric evaluation of the subacromial space in shoulder impingement syndrome. Orthop. Traumatol. Surg. Res. [Internet]. 2022;108(2):103110. 10.1016/j.otsr.2021.103110 10.1016/j.otsr.2021.103110 34649000

[19] Kowalsky MS, Bell J-E, Ahmad CS. Arthroscopic treatment of subcoracoid impingement caused by lesser tuberosity malunion: a case report and review of the literature. J. Shoulder Elbow Surg. [Internet]. 2007;16(6):e10–4. 10.1016/j.jse.2006.09.018 10.1016/j.jse.2006.09.018 17368925

[20] Lädermann A, Chagué S, Preissmann D, Kolo FC, Zbinden O, Kevelham B et al. Acromioplasty during repair of rotator cuff tears removes only half of the impinging acromial bone. JSES Int [Internet]. 2020;4(3):592–600. 10.1016/j.jseint.2020.03.009 10.1016/j.jseint.2020.03.009 PMC7479029 32939492

[21] Lädermann A, Cunningham G, Chagué S, Charbonnier C. Sexual Activities as Risk Factors of Rotator Cuff Lesions: A Prospective Cohort Study. Sex. Disabil. [Internet]. 2018;36(4):305–311. 10.1007/s11195-018-9543-y 10.1007/s11195-018-9543-y PMC6244532 30524154

[22] Lewis JS. Rotator cuff tendinopathy: a model for the continuum of pathology and related management. Br. J. Sports Med. [Internet]. 2010;44(13):918–923. 10.1136/bjsm.2008.054817 10.1136/bjsm.2008.054817 19364757

[23] McCreesh KM, Crotty JM, Lewis JS. Acromiohumeral distance measurement in rotator cuff tendinopathy: is there a reliable, clinically applicable method? A systematic review [Internet]. British Journal of Sports Medicine. 2015;49(5):298–305. 10.1136/bjsports-2012-092063 10.1136/bjsports-2012-092063 25690908

[24] Moor BK, Bouaicha S, Rothenfluh DA, Sukthankar A, Gerber C. Is there an association between the individual anatomy of the scapula and the development of rotator cuff tears or osteoarthritis of the glenohumeral joint? A radiological study of the critical shoulder angle. Bone Joint J. [Internet]. 2013;95-B(7):935–941. 10.1302/0301-620X.95B7.31028 10.1302/0301-620X.95B7.31028 23814246

[25] Neer CS 2. nd. Anterior acromioplasty for the chronic impingement syndrome in the shoulder: a preliminary report. J. Bone Joint Surg. Am. [Internet]. 1972;54(1):41–50. Available from: https://www.ncbi.nlm.nih.gov/pubmed/5054450 5054450

[26] Platzer P, Kutscha-Lissberg F, Lehr S, Vecsei V, Gaebler C. The influence of displacement on shoulder function in patients with minimally displaced fractures of the greater tuberosity. Injury [Internet]. 2005;36(10):1185–1189. 10.1016/j.injury.2005.02.018 10.1016/j.injury.2005.02.018 15963996

[27] Rouleau DM, Yves Laflamme G, Mutch J. Fractures of the greater tuberosity of the humerus: a study of associated rotator cuff injury and atrophy [Internet]. Shoulder & Elbow. 2016;8(4):242–249. 10.1177/1758573216647896 10.1177/1758573216647896 PMC5023050 27660656

[28] Sanguanjit P, Apivatgaroon A, Boonsun P, Srimongkolpitak S, Chernchujit B. The differences of the acromiohumeral interval between supine and upright radiographs of the shoulder. Sci. Rep. [Internet]. 2022;12(1):9404. 10.1038/s41598-022-13632-0 10.1038/s41598-022-13632-0 PMC9174172 35672458

[29] Schneider P, Eberly DH. Geometric Tools for Computer Graphics [Internet]. Elsevier; 2002. Available from: https://play.google.com/store/books/details?id=3Q7HGBx1uLIC

[30] Shinagawa K, Hatta T, Yamamoto N, Kawakami J, Shiota Y, Mineta M et al. Critical shoulder angle in an East Asian population: correlation to the incidence of rotator cuff tear and glenohumeral osteoarthritis [Internet]. Journal of Shoulder and Elbow Surgery. 2018;27(9):1602–1606. 10.1016/j.jse.2018.03.013 10.1016/j.jse.2018.03.013 29731396

[31] Singh H, Thind A, Mohamed NS. Subacromial Impingement Syndrome: A Systematic Review of Existing Treatment Modalities to Newer Proprioceptive-Based Strategies. Cureus [Internet]. 2022;14(8):e28405. 10.7759/cureus.28405 10.7759/cureus.28405 PMC9509002 36171841

[32] Spiegl UJ, Horan MP, Smith SW, Ho CP, Millett PJ. The critical shoulder angle is associated with rotator cuff tears and shoulder osteoarthritis and is better assessed with radiographs over MRI. Knee Surg. Sports Traumatol. Arthrosc. [Internet]. 2016;24(7):2244–2251. 10.1007/s00167-015-3587-7 10.1007/s00167-015-3587-7 25820655

[33] Timmons MK, Lopes-Albers AD, Borgsmiller L, Zirker C, Ericksen J, Michener LA. Differences in scapular orientation, subacromial space and shoulder pain between the full can and empty can tests. Clin. Biomech. [Internet]. 2013;28(4):395–401. 10.1016/j.clinbiomech.2013.01.015 10.1016/j.clinbiomech.2013.01.015 23473974

[34] Via AG, De Cupis M, Spoliti M, Oliva F. Clinical and biological aspects of rotator cuff tears. Muscles Ligaments Tendons J [Internet]. 2013;3(2):70–79. 10.11138/mltj/2013.3.2.070 10.11138/mltj/2013.3.2.070 PMC3711705 23888289

[35] WaTerman BR, Newgren J, Gowd AK, Cabarcas B, Lansdown D, Bach BR et al. Randomized Trial of Arthroscopic Rotator Cuff With or Without Acromioplasty: No Difference in Patient-Reported Outcomes at Long-Term Follow-Up [Internet]. Arthroscopy: The Journal of Arthroscopic & Related Surgery. 2021;37(10):3072–3078. 10.1016/j.arthro.2021.04.041 10.1016/j.arthro.2021.04.041 33940126

[36] Woodmass JM, Al Khatib L, McRae S, Lapner P, Mascarenhas R, Neogi D et al. Arthroscopic Rotator Cuff Repair with and without Acromioplasty in the Treatment of Full-Thickness Rotator Cuff Tears: Long-Term Outcomes of a Multicenter, Randomized Controlled Trial. J. Bone Joint Surg. Am. [Internet]. 2022;104(23):2101–2107. 10.2106/JBJS.22.00135 10.2106/JBJS.22.00135 36476738

[37] Wu G, van der Helm FCT, Veeger HEJD, Makhsous M, Van Roy P, Anglin C et al. ISB recommendation on definitions of joint coordinate systems of various joints for the reporting of human joint motion–Part II: shoulder, elbow, wrist and hand. J. Biomech. [Internet]. 2005;38(5):981–992. 10.1016/j.jbiomech.2004.05.042 10.1016/j.jbiomech.2004.05.042 15844264

[38] Yoo J-S, Heo K, Yang J-H, Seo J-B. Greater tuberosity angle and critical shoulder angle according to the delamination patterns of rotator cuff tear [Internet]. Journal of Orthopaedics. 2019;16(5):354–358. 10.1016/j.jor.2019.03.015 10.1016/j.jor.2019.03.015 PMC6461600 31011247

